# Correlation between Thyroid Responses and Inflammatory Cytokines in Critically Ill COVID-19 Patients

**DOI:** 10.3390/biomedicines11010026

**Published:** 2022-12-22

**Authors:** Albert Figueras Castilla, María A. Ballesteros Vizoso, Amanda Iglesias Coma, Antonia Barceló, Jesús A. Barea-Mendoza, Paula Argente del Castillo, Begoña Guardiola, Jon Pérez-Bárcena, Juan A. Llompart-Pou

**Affiliations:** 1 Servei de Medicina Intensiva, Hospital Universitari Son Espases, 07120 Palma, Spain; 2 Servei d’Anàlisi Clíniques, Hospital Universitari Son Espases, 07120 Palma, Spain; 3 Institut d’Investigació Sanitària Illes Balears (IdISBa), 07120 Palma, Spain; 4 Centro de Investigación Biomédica en Red de Enfermedades Respiratorias, Instituto de Salud Carlos III (CIBERES), 28029 Madrid, Spain; 5 Servicio de Medicina Intensiva, UCI Trauma y Emergencias, Hospital Universitario 12 de Octubre, 28041 Madrid, Spain

**Keywords:** COVID-19, thyroid hormones, cytokines, organ failure

## Abstract

Mechanisms involved in thyroid dysfunction in critically ill coronavirus disease 2019 (COVID-19) patients are not clear. Our objective was to correlate the thyroid response with the pro- and anti-inflammatory cytokines profile in critically ill COVID-19 patients. This was a prospective single-center study. We studied the relationship between continuous variables by using Pearson correlation and simple linear regression. Multiple logistic regression analysis was performed to analyze the factors independently associated with mortality. Seventy-eight patients were included in the study at intensive care unit (ICU) admission and 72 had a measurement of the thyroid and inflammatory profile at day 5. No significant correlations were found between thyroid stimulating hormone (TSH), free triiodothyronine (fT3) and free thyroxine (fT4) and inflammatory cytokines at ICU admission. At day 5, fT4, was inversely correlated with IL-10 (*p* = 0.035). IL-10 was associated with maximum lactate (*p* < 0.001) and SOFA score values (*p* = 0.012). The multiple logistic regression analysis showed that there was a significant relationship between IL-10 (day 5) and in-hospital mortality after adjusting by age and severity of illness. In conclusion, we found that the thyroid hormone profile and inflammatory cytokines had a weak correlation at ICU admission. Associations of interest between fT4 and IL-10 were found at day 5. IL-10 at day 5 was found to be correlated with low fT4 and markers of organ failure and death.

## 1. Introduction

Coronavirus disease 2019 (COVID-19), the disease caused by the severe acute respiratory syndrome coronavirus 2 (SARS-CoV-2) is recognized as a complex disorder affecting many body systems [1,2]. In this context, disturbances of the thyroid hormone responses are common in COVID-19 patients [3,4], since Angiotensin-converting enzyme 2 (ACE2), the functional receptor for SARS-CoV-2, plays a role in the pathogenesis of COVID-19 and is present at hypothalamic and pituitary structures [5] and in the thyroid gland [6]. These disturbances appear proportional to the severity of the disease, being more prevalent in critically ill COVID-19 patients and are associated with outcomes [3,4,5,7,8,9,10]. However, whilst this constitutes a well-known phenomena, the mechanisms involved are less clear [3,4]. One of the main mechanisms proposed includes the inflammatory responses. Different studies have shown that the inflammatory responses constitute a key factor in COVID-19 patients [11,12,13]. Initial studies suggest that the inflammatory cytokines profile responses seem to be associated with the thyroid hormone responses [14,15].

In a previous study including critically ill COVID-19 patients, we observed that thyroid response was commonly affected and free thyroxine (fT4) at day 5 after intensive care unit (ICU) admission was associated with mortality [9]. This finding was consistent with a recent meta-analysis that showed that survivors had higher fT4 levels than non-survivors [10].

To evaluate potential underlying mechanisms, our objective was to correlate the thyroid response with the pro- and anti-inflammatory cytokines profile in critically ill COVID-19 patients.

## 2. Materials and Methods

This was a prospective single-center study involving critically ill COVID-19 patients admitted to the ICU of a tertiary University Hospital (Hospital Universitari Son Espases, Palma, Spain). The study was approved by the Comité Ètic Illes Balears (ref IB 4445/21 PI). Written informed consent was obtained from patients or closest relatives.

### 2.1. Patients

We prospectively studied consecutive adult (≥18 years) critically ill COVID-19 patients admitted to the ICU of our tertiary University Hospital from January to March 2021. Infection was confirmed by means of reverse transcription–polymerase chain reaction (RT-PCR) from nasopharyngeal swab or tracheal aspirate (if intubated). Patients were excluded if they had history of prior hypopituitarism affecting thyroid axis, thyroid gland disease and/or were on anti-thyroid drugs and/or thyroid hormone replacement. All patients received steroids as per the protocol for severe COVID-19 at the time of the study. 

### 2.2. Outcomes

The main outcome measure was the relationship of the thyroid response with cytokines profile in critically ill COVID-19 patients.

### 2.3. Measurement of Thyroid Hormones 

Blood was drawn from a central venous catheter the next morning after ICU admission (07:00 h) and on the 5th day after ICU admission (07:00 h). Blood samples were collected in a serum separator tube and centrifuged (3000 rpm, 10 min). Serum was stored at −80 °C until analysis.

Analyses of serum concentrations of thyroid stimulating hormone (TSH), fT4 and free triiodothyronine (fT3) were performed on ARCHITECT i2000 (Abbott Diagnostics, USA). The ARCHITECT two-step immunoassay uses Chemiluminescent Microparticle Immunoassay (CMIA) technology. On the other hand, the analysis of reverse triiodothyronine (rT3) is measured by competitive radio-immunoassay (RIA). The manufacturer reports a linearity of 0.01–100 µUI/mL for TSH, 0.4–5 ng/dL for fT4 assays, 1.5–20 pg/mL for fT3 assays and 0.09–0.35 ng/mL for rT3 assays. Long-term inter-assay imprecision was satisfactory, with TSH coefficients of variation (CV) of 5.26% at 0.38 µUI/mL and 4.37% at 4.58 µUI/mL, fT4 CV of 3.6% at 0.98 pmol/L and 4.08% at 1.98 pmol/L, fT3 CV of 4.3 at 2.28 pg/mL and 5.12% at 6.05 pg/mL and rT3 CV of 7.4% at 0.28 ng/mL and 5.7% at 0.62 ng/mL. Lower limits of detection for TSH, fT4, fT3 and rT3 were <0.01 µUI/mL, 0.4 ng/dL, 1.5 pg/mL and 0.09 ng/mL, respectively. Reference values for TSH, fT4, fT3 and rT3 were 0.35–4.95 µUI/mL, 0.70–1.48 ng/dL, 1.58–3.91 pg/mL and 0.09–0.35 ng/mL, respectively. The laboratory maintenance was reviewed regularly according to manufacturers’ specifications. 

### 2.4. Measurement of Cytokines

The concentrations of GM-CSF, IFN-γ, IL-1β, IL-2, IL-4, IL-5, IL-6, IL-8, IL-10 IL-12, TNF-α and VEGF in serum were analyzed using a human cytokine magnetic bead panel (RYD-LHSCM000 Luminex Performance Human HS Cytokine Magnetic Panel A (12-Plex), R&D Systems, Minneapolis, MN, USA) according to the manufacturer’s instructions. A total of 50 µL of each sample, 50 µL of assay buffer and 25 µL of premixed beads were added to the sample wells. The plate was incubated with shaking overnight (16–18 h) at 4 °C. The next day, it was washed three times, and 50 µL of Biotin-Antibody Cocktail were added to each well. Samples were incubated with shaking for 1 h at room temperature, and afterward, 50 µL of Streptavidin-PE were added to each well. Samples were then incubated with shaking for 30 min at room temperature. Samples were finally washed three times and 100 μL of Wash Buffer was added to all wells, and the plate was read in a MAGPIX^®^ (Luminex, Austin, TX, USA). Assay sensitivity according to the manufacturer was as follows: 0.155 pg/mL for GM-CSF, 0.25 pg/mL for IFN-γ, 0.146 pg/mL for IL-1β, 0.13 pg/mL for IL-2, 1.14 pg/mL for IL-4, 0.06 pg/mL for IL-5, 0.14 pg/mL for IL-6, 0.04 pg/mL for IL-8, 0.211 pg/mL for IL-10, 1.81 pg/mL for IL-12, 0.29 pg/mL for TNF-α and 0.88 pg/mL for VEGF.

### 2.5. Statistical Analysis

Categorical data are expressed as numbers and percentages. Continuous variables are shown as mean ± standard deviation (SD) or median and interquartile range (IQR), as appropriate. Differences between groups were compared using the independent Student T test or the Wilcoxon test (for continuous variables) and chi-square or Fisher’s exact test (for categorical variables), as appropriate. We studied the relationship between continuous variables using Pearson correlation and simple linear regression. Multiple logistic regression analysis was performed to analyze the factors independently associated with mortality. The variables entered in the logistic regression analysis were those significantly associated with mortality in the univariate analysis. The model was evaluated by calculating the receiver operating characteristics (ROCs),the area under the curve (AUC) and the Hosmer–Lemeshow test. A *p* value < 0.05 was considered statistically significant. Data analysis and plots were programmed using Rstudio 2022 (RStudio, PBC, Boston, MA, USA).

## 3. Results

During the study period, 84 critically ill COVID-19 patients were admitted to the ICU. Six were excluded from the analysis as they had previous thyroid disease receiving thyroid hormone replacement. Overall, 78 patients were included in the study at ICU admission and 72 had a measurement of the thyroid and inflammatory profile at day 5. Six patients died in the ICU before obtaining the second sample. Baseline clinical characteristics of the 78 critically ill COVID-19 patients are shown in Table 1. 

Specific treatments received during ICU stay are summarized in Table 2. 

**Figure 1:**
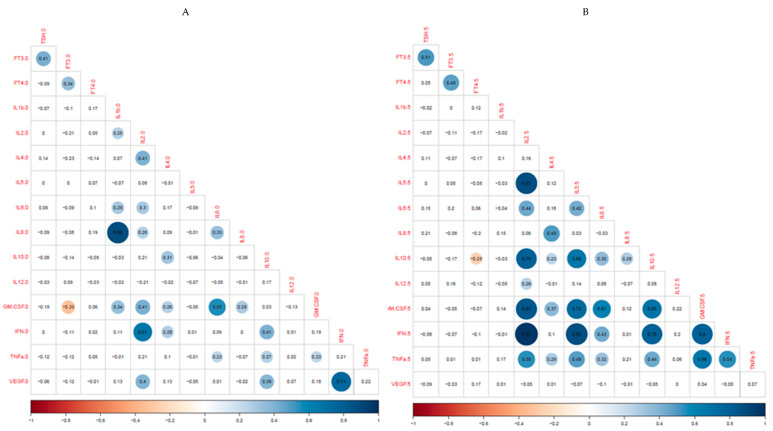
Heat map showing the associations of the thyroid hormones and cytokines evaluated at ICU admission (**A**) and day 5 (**B**). Pearson correlation coefficients are displayed. Circles representing the strength of correlation are omitted if they do not achieve statistical significance. Blue and red circles represent positive and negative correlations.

The nature of the associations between thyroid hormones and cytokines at ICU admission and day 5 are summarized in Figure 1. Associations were found to be stronger on day 5.

**Figure 2:**
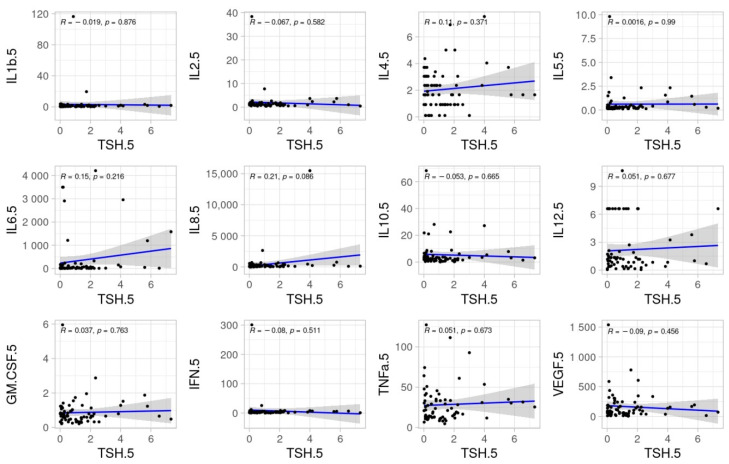
Scatter dots of correlations between TSH and cytokines at day 5.

**Figure 3:**
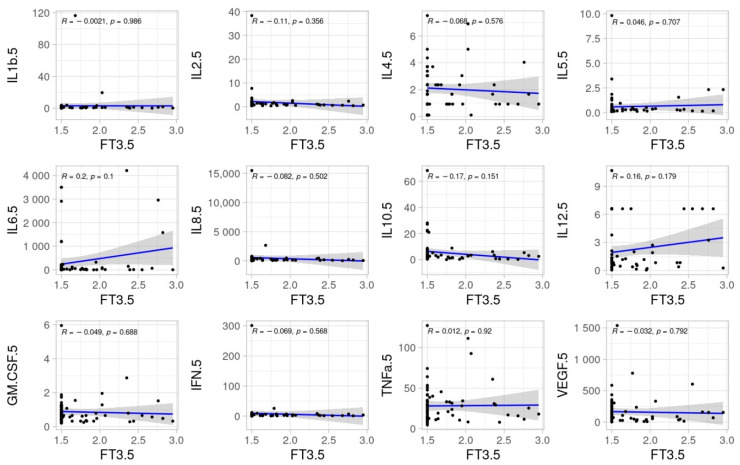
Scatter dots of correlations between fT3 and cytokines at day 5.

**Figure 4:**
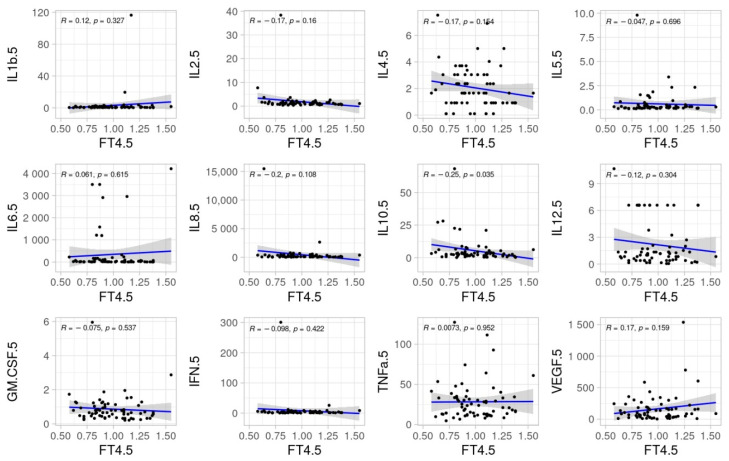
Scatter dots of correlations between fT4 and cytokines at day 5.

No significant correlations were found between TSH, fT3 and fT4 and inflammatory cytokines at ICU admission. The pairwise relationships of TSH, fT3, and fT4 with inflammatory cytokines at day 5 are shown in Figure 2, Figure 3 and Figure 4.

**Figure 5:**
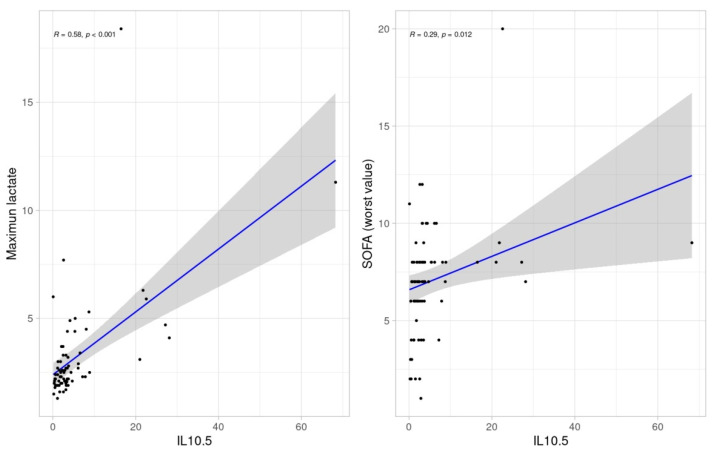
Scatter dots of correlations between IL-10 at day 5 and SOFA score values and maximum lactate.

At day 5, fT4, was inversely correlated with IL-10 (*p* = 0.035). In the same direction, IL-10 was associated with maximum lactate (*p* < 0.001) and SOFA score values (*p* = 0.012) (Figure 5).

Finally, the multiple logistic regression analysis showed that there was a significant relationship between IL-10 (day 5) and in-hospital mortality after adjusting by age and severity of illness, stated by the SOFA score and the worst paO2/FiO2 (Table 3). 

## 4. Discussion

Our study showed that the thyroid hormone profile and inflammatory cytokines had a weak correlation in critically ill COVID-19 patients at ICU admission. Specifically, associations of interest between fT4 and IL-10 were found at day 5 rather than on ICU admission. IL-10 at day 5 was found to be correlated with markers of organ failure and death. 

In our previous study with critically ill COVID-19 patients [9], we observed that thyroid response was commonly affected and fT4 at day 5 after ICU admission was associated with mortality [9]. This finding was consistent with a recently published meta-analysis [10]. However, since its underlying mechanisms are not clear, different authors supported that determining whether thyroid impairment truly impacts outcomes or in turn, is a marker of severity, remains inconclusive [16]. In this line, the objective of this follow-up study was to evaluate the correlation of inflammatory cytokines and the thyroid response. Interestingly, associations were weak at ICU admission and stronger in the follow-up. Specifically, fT4 at day 5, which was associated with mortality in our previous study [9], had a significant inverse relationship with IL-10 levels (*p* = 0.035). Low levels of fT4, and therefore, high IL-10, at day 5 were indeed correlated with maximum lactate and SOFA scores, suggesting an inflammatory pathway to organ failure and death. This is consistent with recent evidence suggesting that changes in thyroid hormones in critically ill COVID-19 patients are mediated by the inflammatory responses [14,15,17]. 

Contrary to our previous study [9], after including the inflammatory cytokines measurements in the model, fT4 lost its prognostic value in favour of IL-10 at day 5. Taken together, these findings suggest that IL-10 could play a role in thyroid hormones regulation and could act as a trigger for thyroid dysfunction and organ failure (14). Previous studies have also shown the potential role of IL-10 in COVID-19 pathogenesis and as predictor of severity [13]. Moreover, blockage of IL-10 has been postulated as a target for treatment of severe COVID-19 patients [17]. Lu et al. suggest that as endogenous IL-10 production increases it might function as an immune activating/proinflammatory agent stimulating the production of other mediators of the cytokine storm and could also amplify the viral sepsis-related hyperinflammation observed in critically ill COVID-19 patients [18,19]. This is consistent with the relationship found between IL-10 and maximum lactate and SOFA scores. 

The strengths of our study included the follow-up data with two evaluations and the analysis of a broad range of cytokines and thyroid hormones. Longitudinal analysis to demonstrate the dynamics of cytokine and thyroid hormones associated with disease progression could help to clarify the mechanisms of these associations, and therefore, guide more effective interventions in patients with severe COVID-19. 

Our study has some limitations. First, we studied a relatively small (but homogeneous) sample of critically ill COVID-19 patients. Second, steroids, which are well-known agents that can affect thyroid and inflammatory responses by up- or down-regulating IL production, were a standard of care and used in all patients at the time the study was performed [20]. However, since the same dose and protocol was applied to all patients in our institution (methylprednisolone 125 mg daily for 3 days followed by dexamethasone 6 mg daily for 7 days) this confounding factor was minimized and its therefore unlikely that our results could be influenced by different steroid doses. 

## 5. Conclusions

We found that the thyroid hormone profile and inflammatory cytokines had a weak correlation in critically ill COVID-19 patients at ICU admission. Associations of interest between fT4 and IL-10 were found at day 5 after ICU admission. IL-10 at day 5 was found to be correlated with lowfT4 and markers of organ failure and death.

## Figures and Tables

**Table 1:** Baseline clinical characteristics of the critically ill COVID-19 patients included in the study.

	N: 78
Female, *n* (%)	23 (29.5%)
Age (years)	62.1 (12.4)
SAPS II	40.6 (14.2)
APACHE II	18.0 (8.0)
Admission SOFA	5.4 (2.5)
**Comorbidities, *n* (%)**	
Hypertension	43 (55.1%)
Diabetes mellitus	20 (25.6%)
Dyslipidemia	37 (47.4%)
COPD	10 (12.8%)
Chronic kidney disease	9 (11.5%)
Chronic cardiac disease	12 (15.4%)
Malignancy (active)	9 (11.5%)
**Prior treatment, *n* (%)**	
Beta-blockers	13 (16.6%)
ACE inhibitors	17 (21.8%)
ARBs	19 (24.3%)
Statins	30 (38.4%)

SAPS II: Simplified Acute Physiology Score II; APACHE II: Acute Physiology And Chronic Health Evaluation II; SOFA: Sepsis related Organ Failure Assessment; COPD: Chronic Obstructive Pulmonary Disease; ACE: Angiotensin-converting enzyme; ARBs: Angiotensin receptor blockers.

**Table 2:** Treatments received in the ICU and outcomes.

**Treatment in the ICU**
Steroids: 78 (100%)
Remdesivir: 11 (14.1%)
Tocilizumab: 15 (9.2%)
Prophylactic heparin: 43 (55.1%)
Intermediate dose heparin: 28 (35.9%)
Full anticoagulation: 7 (8.9%)
Mechanical ventilation: 67 (85.9%)
V-V ECMO: 5 (6.4%)
**Outcomes**
ICU LOS (days): 31.1 (31.5)
In-hospital LOS (days): 46.1 (38.7)
In-hospital Mortality: 23 (29.5%)

V-V ECMO: Veno-Venous Extracorporeal Membrane Oxygenation; ICU: Intensive Care Unit; LOS: Length Of Stay.

**Table 3:** Logistic Regression Analysis—in-hospital mortality.

Variable	OR (95% CI)	*p* Value
Age	1.11 (1.02–1.23)	0.034
SOFA	1.49 (0.98–2.46)	0.081
PaO2/FiO2	0.96 (0.91–0.99)	0.018
IL-10 (day 5)	1.23 (1.05–1.63)	0.040

Values in parentheses are 95 per cent confidence intervals, Variables with *p* < 0.10 in univariate analysis were entered into multivariable models, Area under receiver operating characteristic curve (AUROC 0.92), Hosmer–Lemeshow (HL) χ^2^ = 15.74, *p* = 0.07.

## Data Availability

Data used in this study can be obtained from the corresponding author upon reasonable request.
